# Acceptability and satisfaction with *emma*, a smartphone application dedicated to suicide ecological assessment and prevention

**DOI:** 10.3389/fpsyt.2022.952865

**Published:** 2022-08-11

**Authors:** Margot Morgiève, Daniel Yasri, Catherine Genty, Jonathan Dubois, Marion Leboyer, Guillaume Vaiva, Sofian Berrouiguet, Jérôme Azé, Philippe Courtet

**Affiliations:** ^1^Université Paris Cité, CNRS, Inserm, Cermes3, Paris, France; ^2^Department of Emergency Psychiatry and Acute Care, Lapeyronie Hospital, CHU Montpellier, Montpellier, France; ^3^ICM – Paris Brain Institute, Hôpital de la Pitié-Salpêtriére, Paris, France; ^4^GEPS – Groupement d’Étude et de Prévention du Suicide, Paris, France; ^5^Fondation Fondamental, Hôpital Albert-Chenevier, Créteil, France; ^6^Faculté de Médicine, Institut National de la Santé et de la Recherche Médicale, Université Paris-Est Créteil, Créteil, France; ^7^Assistance Publique Hôpitaux de Paris, Pôle de Psychiatrie et Addictologie, Hôpitaux Universitaires Henri Mondor, Créteil, France; ^8^CHU Lille, Hôpital Fontan, Department of Psychiatry, Lille, France; ^9^Centre National de Resources and Résilience pour les Psychotraumatisme, Université de Lille, Lille, France; ^10^CNRS UMR-9193, SCALab - Sciences Cognitives et Sciences Affectives, Université de Lille, Lille, France; ^11^Laboratoire du Traitement de l’Information Médicale, INSERM UMR1101, CHRU Brest, Brest, France; ^12^LIRMM, CNRS, Univ Montpellier, Montpellier, France

**Keywords:** mHealth, ecological momentary assessment (EMA), ecological momentary intervention (EMI), suicide, prevention, acceptability, satisfaction, smartphone application

## Abstract

**Background:**

As mHealth may contribute to suicide prevention, we developed *emma*, an application using Ecological Momentary Assessment and Intervention (EMA/EMI).

**Objective:**

This study evaluated *emma* usage rate and acceptability during the first month and satisfaction after 1 and 6 months of use.

**Methods:**

Ninety-nine patients at high risk of suicide used *emma* for 6 months. The acceptability and usage rate of the EMA and EMI modules were monitored during the first month. Satisfaction was assessed by questions in the monthly EMA (Likert scale from 0 to 10) and the Mobile App Rating Scale (MARS; score: 0–5) completed at month 6. After inclusion, three follow-up visits (months 1, 3, and 6) took place.

**Results:**

Seventy-five patients completed at least one of the proposed EMAs. Completion rates were lower for the daily than weekly EMAs (60 and 82%, respectively). The daily completion rates varied according to the question position in the questionnaire (lower for the last questions, LRT = 604.26, df = 1, *p*-value < 0.0001). Completion rates for the daily EMA were higher in patients with suicidal ideation and/or depression than in those without. The most used EMI was the emergency call module (*n* = 12). Many users said that they would recommend this application (mean satisfaction score of 6.92 ± 2.78) and the MARS score at month 6 was relatively high (overall rating: 3.3 ± 0.87).

**Conclusion:**

*Emma* can target and involve patients at high risk of suicide. Given the promising users’ satisfaction level, *emma* could rapidly evolve into a complementary tool for suicide prevention.

## Highlights

- Suicidal risk assessment is a major issue for high-risk patients.
- Assessments in ecological conditions are highly valuable to clinicians.
- Mobile applications allow for an easy way to gather data with high resolution.
- Satisfaction, acceptability, and usage of these novel assessment methods must be determined.
- Depressed and suicidal patients seem to be sensitive to this approach.

## Introduction

### Context

With 800,000 suicides every year and 20-fold more suicide attempts worldwide, suicidal behavior represents a major public health issue (1). Prevention is hampered by the complexity and the number of factors that lead to suicide or non-suicidal self-injury (2). Among the major predictors of death by suicide, the most prominent is a previous attempt, followed by non-suicidal self-injury (3). Moreover, suicidal ideation is almost always present before a suicide attempt, regardless of the social and demographic background (4, 5). In everyday practice, physicians are often faced with the need to identify the most at-risk subjects. Indeed, up to 40% of suicide victims had seen a general practitioner within 1 month before the act, and up to 80% within the previous year (6). To develop effective prevention interventions that should be both pragmatic and innovative, it is essential to understand the psychological mechanisms involved in the development of suicidal ideation and in the translation of these ideas into suicidal acts (7).

### The potential of mHealth for suicide prevention

The fast pace of the digital revolution during the last 10 years paved the way for innovation in the mental health field (8–11). Mobile health (mHealth; defined by the World Health Organization *“as the use of mobile and wireless technologies to support the achievement of health goals”*) (12) offers various possibilities for diagnosis, treatment, remote monitoring, data collection, therapeutic education, and training tools. One of its key advantages is to promote networking between patients and clinicians. This can help to increase access to health services and adherence to treatment (13) and to reduce costs by decreasing the need for hospitalization and intensive care (14). Smartphones, sensors, wearables, big data, machine learning, and other forms of digital technology should be considered to improve suicide risk assessment and suicide prevention (15, 16).

### Ecological momentary assessment

Smartphones allow people to self-monitor and self-manage through a completely new approach that is very different from the classical face-to-face interviews and paper-based assessments. Ecological Momentary Assessment (EMA), which uses repeated sampling in the natural environment and in real time (17), has an important part to play in this context. Using EMAs, a high number of observations can be collected over time, even several times per day, thus reducing the biases associated with retrospective data collection (18) and increasing the ecological validity and generalizability by avoiding “laboratory bias.” These multiple assessments allow more complex and nuanced research and higher statistical power, better highlighting the dynamic associations between the observed processes.

Previous studies showed that EMA for assessing suicidal ideas does not have iatrogenic effects, and it is a safe way to monitor suicidal thoughts (19, 20). Moreover, people are more likely to disclose sensitive information about their lives online or on a mobile application than in real-life interviews (21). Therefore, regular longitudinal data collection can provide a more accurate picture of the emotional and cognitive context in which suicidal thoughts appear.

### From ecological momentary assessment to ecological momentary intervention

Real-time monitoring also allows ecological momentary interventions (EMI) at the time of suicidal crisis, although this approach has been underused (22). Evidence-based clinical guidelines have already been proposed for the development of applications to prevent suicidal behavior and include: mood and suicidal thought tracking, development of a safety plan, recommendation of activities, information and education, access to support networks, access to emergency counseling, information trustworthiness, and an online help functionality in the event of a suicidal crisis, which is the most effective strategy for the prevention of suicidal behavior, including links with relatives (23). Although most mobile applications for suicide prevention offer similar functionality, few meet all these recommendations (23). In fact, despite an increasing number of mHealth research in suicidology during the past decade, it faces many legal (e.g., patient data storage), clinical (e.g., monitoring and managing risk at the same time), and methodological obstacles that *emma* aims to solve. The application meets all the ethical and regulatory standards imposed by the European Data Protection Regulations; it provides real-time clinical tools by proposing EMIs based on response thresholds to ecological momentary assessments (EMA); it was built on a participatory co-design methodology that considers the needs of patients, data from the scientific literature, and good practice guidelines in suicidology.

The *emma* application was thus developed in accordance with these clinical guidelines to finely assess the patient’s emotions, thoughts, suicidal ideation, behaviors, and their context of occurrence through EMAs (daily, weekly, monthly, and spontaneous). Using an algorithm that will be later discussed, data were collected through these EMAs and thresholds were designed depending on the patient’s answers to some critical questions leading to *emma* automatically proposing adapted EMIs categorized into modules (24). The first step of our study is to assess whether patients provide the information that is intended to be collected through repeated high-resolution assessments under ecological conditions. As *emma* uses these fine grind assessments, we also aim to develop further studies focusing on improving the detection of suicidal acts imminence. The EMA/EMI combination should be particularly appropriate during the first weeks after discharge from the emergency department following a suicidal crisis when patients are at very high risk of suicide.

### Objective

The main objective was to evaluate the usage rate and acceptability of the *emma* application in the first month, and satisfaction after 1 month and after 6 months of utilization to better assess the global satisfaction of the application, both on the medical and the technological side.

This first step of characterization of the way in which the patients complete the information *via* the repeated evaluations with high resolution under ecological conditions will make it possible a second time to analyze the evolution of their contents more finely.

## Materials and methods

### Study design

This is a prospective, longitudinal, and multicentric research study proposed for patients followed in psychiatric consultation or received in psychiatric emergencies. The inclusion sites were composed of four university hospitals centers (Brest, Montpellier, Lille, Créteil) across France (number: NCT03410381, 18/01/2018),^1^ authorized by the French Health Ministry (ANSM, 30/11/2017), and approved by the Est IV Ethical Committee for the Protection of Patients (10/10/2017).

### Participants

In total, 100 participants were included providing sufficient headcount to implement predictive models, which is the ultimate goal of the study. Inclusion criteria were: ≥18 years of age, suicidal ideation, or suicide attempt in the last week (score ≥ 2 out of 3 for the suicide item of the Inventory of Depressive Symptoms-Clinician Rated-30, IDSC-30, and the Columbia–Suicide Severity Rating Scale, CSSRS), as well as the ability to understand the study nature, purpose, and methodology, and owning an Android or iOS smartphone. Exclusion criteria were: refusal to participate, being under guardianship or curatorship, being deprived of liberty by administrative decision, not being affiliated to a social security scheme, being in a period of exclusion in relation to another protocol, and inability to understand and/or answer questionnaires. Participants did not receive any compensation for their participation in the study.

### Intervention

The clinical protocol that enabled the implementation of *emma* has been extensively described in a previous article (24). The present study focused on the participants’ clinical characteristics assessed by specifically trained psychologists, with several years of experience in clinical research leading to consistent measures across the study, at the inclusion visit (month 0, M0), utilization of the EMAs and EMIs present in the application during the first month, and satisfaction with *emma* at the M1 (through *emma*) and M6 visits. This subset of variables was chosen as the dropout rate after the first month had not allowed any statistically accurate analysis for the daily, weekly, and monthly EMAs, while the 6-month satisfaction evaluation being in person at the follow-up visit had enough data.

At M0 (inclusion visit), clinical diagnoses of DSM-5 psychiatric disorders were made with the Mini-International Neuropsychiatric Interview (25). Suicidal thoughts and depression severity were assessed using the self-report Quick Inventory of Depressive Symptomatology (QIDS) and the IDSC-30 (26) at the M0 and M1 visits. Suicidal ideation was assessed with the suicidal ideation items of the Columbia-Suicide Severity Rating Scale (CSSRS-SI) (score from 0 to 5) (27). All the self-administered evaluations are standard scales already evaluated in the literature with a larger sample size and the more general population. Satisfaction with the application was measured with satisfaction questions in the monthly EMA *via emma* at M1 and with the Mobile App Rating Scale (MARS) (28) at M6. Data on education level and social media use (Twitter, Facebook, or Instagram accounts) were collected at inclusion.

### *Emma*’s content

**FIGURE 1:**
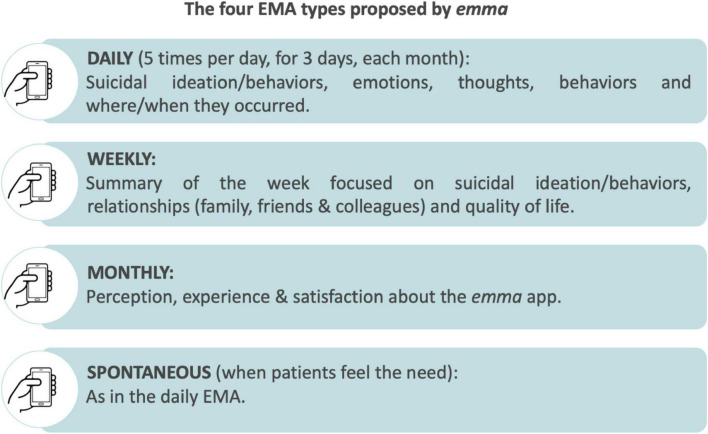
*Emma*’s assessments.

*Emma* (Figure 1) proposes different observations to finely assess the patient’s emotions, thoughts, suicidal ideation, behaviors, and contexts of appearance, through,

● Daily assessments (five times a day for three consecutive days, every month) focusing on suicidal ideation/behaviors, emotions, thoughts, and where/when they occurred;

● Weekly evaluations (every Sunday) being a summary of the week focused on suicidal ideation/behaviors, relationships, and quality of life;

● Spontaneous questionnaires that patients can complete whenever they feel the need being the same as the daily EMA;

● Every month, participants answered questions to assess the usefulness, global experience, and level of satisfaction with this application.

**FIGURE 2:**
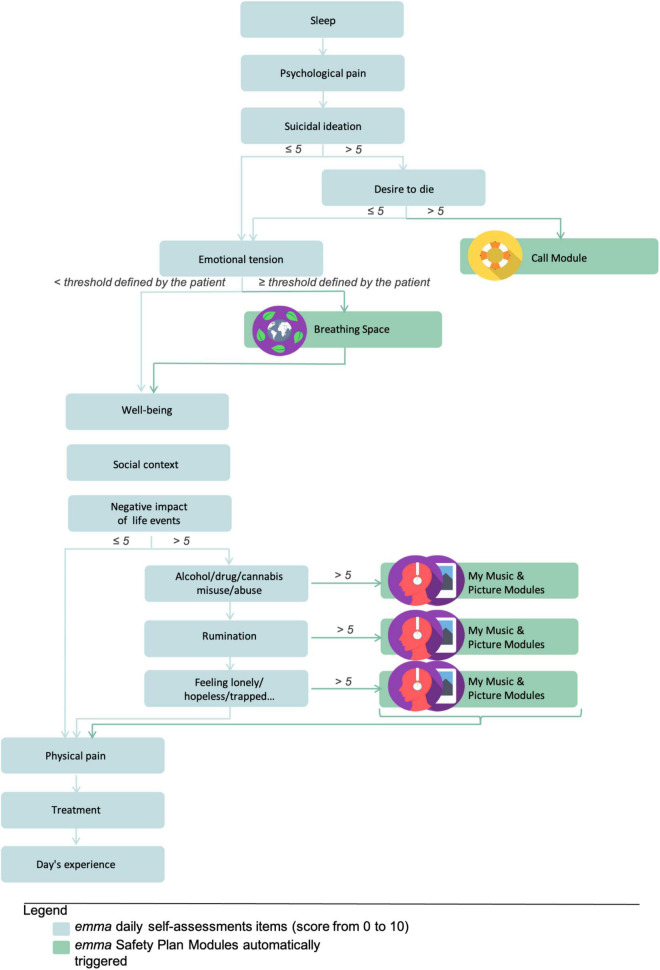
Algorithm for automatic ecological momentary intervention (EMI) triggering according to the ecological momentary assessment (EMA) answer thresholds.

Depending on the patient’s answers and certain thresholds, *emma* proposes different customizable EMI (Figure 2) also categorized into modules.

● A “Call Module” gives the patient access to three different contacts depending on the severity of his condition: (i) the patient’s relatives that he has identified as resource persons; (ii) the department of psychiatric emergencies that follows the patient; and (iii) the SAMU, which is the French Emergency Medical Service available every day at any time of the day or night.

● The “Coping Strategies Module” and “Emotion Regulation Module” allow patients to listen to mindfulness audio guidance, and to list and consult their distraction activities, favorite places, or occupations.

These *personalized safety plan* modules can easily be accessed in need in case of crisis.

### *Emma*’s conception and development

The *emma* application was developed from scratch using the “Security by design” and “Ethic by design” methods to ensure compliance with ethical and data security standards from the design stage. A Privacy Impact Assessment (PIA) has been set up to demonstrate the implementation of privacy protection principles for participants. From a legal and regulatory point of view, the development of the application has also been carried out in compliance with the European General Data Protection Regulation (GDPR) law and the National Commission for Information Technology and Civil Liberties (CNIL) of France and approved by a committee for the protection of individuals. In accordance with the “Privacy Model for Mobile Data Collection Applications (PM-MoDaC)” proposed by Beierle and colleagues, users are informed, before installing the application, about what data are collected and for what purpose; they also have the explicit possibility of opting out (29).

From the early stages of the app’s design, suicidal people were involved as co-researchers in the multidisciplinary team to ensure that the digital tool best met their wishes and needs. A 3-month beta-testing phase then allowed for iterative integration of their feedback before the development of a final version developed for the two major operating systems on the market (IOS and Android) to be proposed to a maximum of individuals and to avoid socio-economic biases (e.g., the first system is much more expensive than the second).

### Primary outcomes

The primary outcomes were the assessment of *emma* acceptability and utilization rate (EMA and EMI use) during the first month, and satisfaction assessed by (i) the first monthly EMA *via emma* (Likert scale from 0 to 10) and (ii) the MARS (score 0–5) at M6.

### Analysis

Participants were classified and described into two groups: (i) patients who quit early before any connection to the application or patients who connected to the application at least once, but never answered any assessment, and (ii) patients who answered at least once to one of the daily, weekly or monthly assessments. In each group, socio-demographic status, depression severity, and suicidal behavior were evaluated at M0 and M1, and visits to the emergency department or to their psychiatric care center were recorded during the first month of follow-up. Quantitative and qualitative variables were described as mean and standard deviation (SD), and numbers and frequencies, respectively. Group differences were tested using the *F*-test for quantitative variables, and the Chi2 or Fisher test (if the expected numbers per group in the Chi2 test was <5) for qualitative data.

Completion rate if not stated otherwise was the proportion of questions in an EMA that the patient answered. The association of patients’ variables with the daily/weekly EMA completion rates was analyzed using univariate mixed effect logistic regression models to fit the probability that a question would be answered in the function of each of these characterizing variables. Individual random intercepts were included to account for the intra-individual correlation. To untangle potential confounding effects on the daily EMA completion rate, a multivariate model was also used with the variables that showed a significant association in the univariate analysis. To avoid collinearity problems, we chose to include only the QIDS score in the multivariate model rather than the presence of MDD at baseline. Odds ratios (OR) and their 95% confidence intervals (CI) were computed and the Likelihood-ratio test (LRT) was used to evaluate the significance of associations.

Logistic mixed regression models were also used to assess the probability of a question being answered according to its position in the daily or weekly EMA.

The sample size was conditioned by a more global question, trying to predict suicidal behavior during the follow-up with ecological momentary assessment data. According to the observed empirical ratio of 1 patient with suicidal relapse during a 6 months follow-up for 4 patients without (in psychiatric hospital departments), 100 patients were found to be a satisfying sample size.

## Results

### Baseline analysis

**FIGURE 3:**
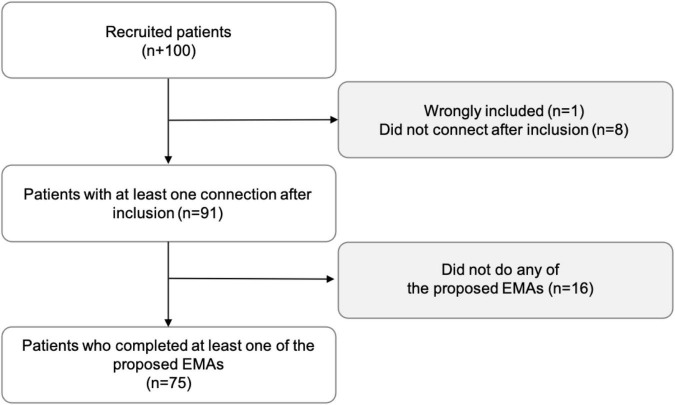
Flowchart of patients’ inclusion.

From May 2018 to January 2020, 100 patients agreed to participate. Reasons for refusal varied, but they were mostly related to the length of the study protocol and follow-up. One patient was wrongly included, 91 connected to the application at least once after inclusion, and 75 completed at least once daily (meaning one out of the 15 proposed during the first 3 days of the month), weekly, or monthly EMA (Figure 3). The participation highly decreased after the first month, especially for the daily EMA. Only 17 (62% dropout) and 58 (12% dropout) patients answered at least once to the daily and weekly EMA during the second month. *Emma* users were mostly women (75%), their mean age was 30 years, and recent suicide attempters (≤8 days) represented roughly one-third of the sample. Sociodemographic characteristics, depression severity, and suicidal features at inclusion were comparable between patients who completed at least one EMA and those who did not (Table 1).

**TABLE 1:** Sociodemographic characteristics, depression severity, and suicidal ideation/behavior.

	Patients who did not complete any EMA (*n* = 24)	Patients who filled in at least one EMA (*n* = 75)	*P*-value
Mean age (SD, min-max)	32.39 (13.12, 18–59)	29.27 (11.02, 18–57)	0.26
Women (n,%)	17 (73.9%)	57 (76%)	1
Mean education level, in years* (SD, min-max)	12.96 (2.08, 9–18)	13.19 (2.01, 9–18)	0.65
IDSC-30 score at inclusion, mean (SD)	40.47 (11.26)	36.41 (11.3)	0.19
QIDS score at inclusion, mean (SD)	19.05 (3.74)	16.9 (5.45)	0.1
Suicide attempters in the last 8 days (n,%)	5 (20.8%)	26 (34.7%)	0.31
Patient with suicidal ideation in the last 8 days (CSSRS-SI mean score ± SD)	16 (3.63 ± 1.77)	71 (3.89 ± 1.231)	0,47

*12 years corresponds to the end of high school in France.

### *Emma* acceptability and utilization (ecological momentary assessment and ecological momentary intervention) during the first month

Among the 91 patients who used the application at least once, 64 went to the M1 follow-up visit. During the first month, 13 patients were admitted to the emergency department for suicidal thoughts and/or suicide attempts.

The following analyses were based on the 75 patients who completed at least one of the proposed EMAs.

**FIGURE 4:**
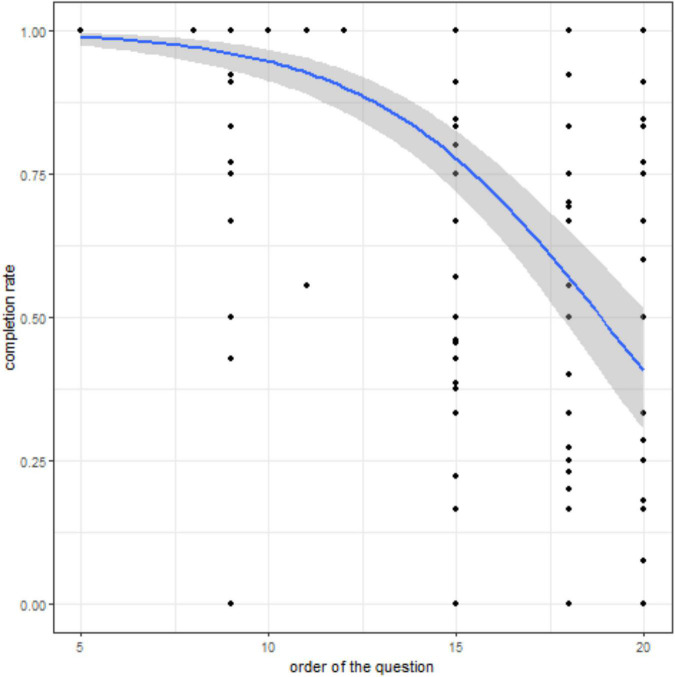
Completion rate according to the question position in the daily EMA. Relationship between the question ranking in the daily EMAs and the completion rate. The predicted probability (blue curve) from the logistic mixed model and its 95% CI (shaded area) are shown. Black dots indicate the completion rate for each patient (*n* = 45) and each question asked but not necessarily answered.

**FIGURE 5:**
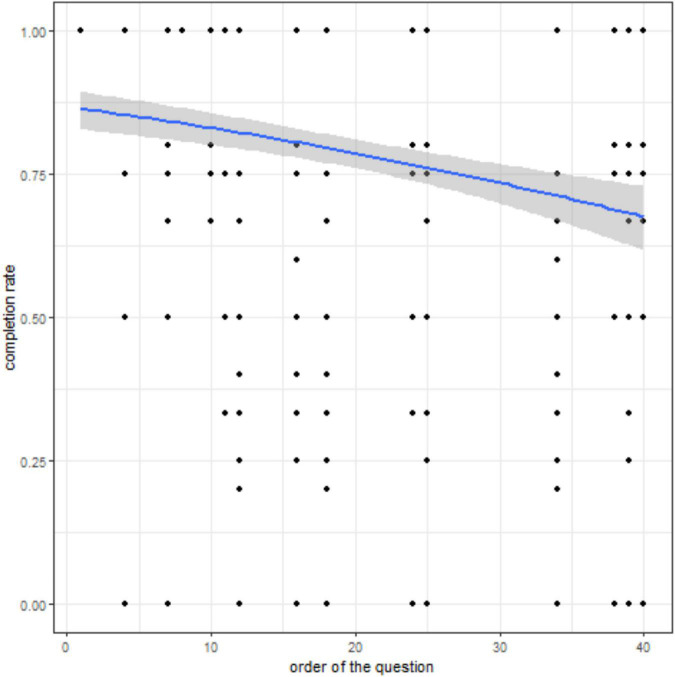
Completion rate according to the question position in the weekly EMA. Relationship between the question ranking in the weekly EMAs and the completion rate. The predicted probability (blue curve) from the logistic mixed model and its 95% CI (shaded area) are shown. Black dots indicate the completion rate for each patient (*n* = 62) and each question asked but not necessarily answered.

During the first month, 45 (60%) patients completed at least one question of the daily EMAs (mean completion rate ± SD, 0.83 ± 0.09). Some patients could have never answered a specific question; consequently, the completion rate can be 0 for a specific question for a given patient. Completion rates according to the question position in the questionnaire (Figure 4) showed a progressive decrease in the answer rate in function of the question number. In the daily EMAs (total number of questions = approximately 20, certain questions being asked only according to the threshold of answers) (LRT = 604.26, df = 1, *p*-value < 0.0001), the completion rate after question 18 fell under 50%. However, in the weekly EMA (total number of questions = approximately 44, certain questions being asked only according to the threshold of answers) (LRT = 57.814, df = 1, *p*-value < 0.0001), the completion rate remained higher than 65% at question 40 (Figure 5). The percentage of patients who filled in the first weekly EMA (*n* = 62; 82.6%) was higher than for the daily EMAs (mean completion rate ± SD, 0.79 ± 0.13). However, this percentage progressively decreased to 64% (*n* = 48) in the second week (mean completion rate ± SD, 0.82 ± 0.09) and to 41 and 39 patients for weeks 3 and 4, respectively. The monthly EMA was filled in by 45 (60%) patients at M1, with a mean completion rate of 0.45 ± 0.42.

Completion rates were significantly different depending on the patient’s clinical characteristics (Table 2). These differences were observed particularly for the daily EMA and tended to disappear for the weekly EMA. Completion rates (mean completion rate ± SD) of the daily, but not the weekly EMA, were higher for women than men (men: 0.80 ± 0.10, women: 0.83 ± 0.09). The daily EMA completion rates were positively associated with depression severity at inclusion (QIDS and IDSC-30 score). Some Odds Ratios for continuous variables seem small but have to be grasped according to the scale of the variables. For example, according to the estimated effect of depression on daily completion (Odds *R* = 1.013 IC = 1.003–1.023; Table 2), passing from mild depression to moderate depression (e.g., passing from 10 to 15 in QIDS score) involves a completion increase of approximately 6.5%. The completion rate of the daily EMA was higher in patients with suicidal ideation at inclusion (0.83 ± 0.9 vs. 0.77 ± 0.14 in patients without suicidal ideation) but was not different among participants who attempted suicide, or not in the last 8 days. Conversely, the weekly EMA completion rate was higher in suicide attempters.

**TABLE 2:** Clinical characteristics and completion rate of the daily and weekly questionnaires.

Clinical characteristic	Daily questionnaires		Weekly questionnaires	
	Odd ratio and 95% CI	*P*-value	Odd ratio and 95% CI	*P*-value
Women	1.605 (1.251–2.049)	0.0002	1.195 (0.983–1.449)	0.07
Depression (IDSC30 score at inclusion)	1.013 (1.003–1.023)	0.009	1.003 (0.995–1.011)	0.43
Depression (QIDS score at inclusion)	1.048 (1.031–1.065)	<0.0001	0.999 (0.984–1.014)	0.89
Depression (IDSC30 score at M1)	1.016 (1.008–1.024)	0.0002	0.999 (0.992–1.006)	0.72
Depression (QIDS score at M1)	1.033 (1.016–1.05)	0.0001	1.001 (0.986–1.016)	0.94
Suicidal ideation (QIDS score at inclusion)	1.522 (1.228–1.887)	0.0001	1.073 (0.904–1.274)	0.42
Suicidal ideation (CSSRS score at inclusion)	2.069 (1.478–2.861)	<0.0001	1.487 (1.047–2.094)	0.03
Suicide attempt in the last 8 days	0.806 (0.646–1.006)	0.06	1.237 (1.029–1.491)	0.02
Lifetime suicide attempt	1.136 (0.896–1.435)	0.29	0.973 (0.713–1.183)	0.79
Major depressive episode	1.304 (0.984–1.712)	0.06	0.936 (0.713–1.215)	0.63
Major depressive disorder	1.295 (1.023–1,649)	0.03	1.175 (0.986–1.403)	0.07
Bipolar disorder	2.107 (1.446–3.178)	0.0002	0.874 (0.705–1.09)	0.23
Type 1 bipolar disorder	1.186 (0.661–2.32)	0.59	0.777 (0.589–1.034)	0.08
Type 2 bipolar disorder	2.639 (1.658–4.471)	0.0001	1.104 (0.809–1.534)	0.54
Current general anxiety disorder	0.718 (0.578–0.893)	0.003	0.911 (0.763–1.089)	0.3
Lifetime panic disorder	1.762 (1.365–2.297)	<0.0001	1.066 (0.89–1.28)	0.49
Actual PTSD	1.503 (0.961–2.47)	0.09	0.948 (0.716–1.27)	0.71
Alcohol use disorder (12 month)	0.927 (0.607–1.465	0.73	1.006 (0.799–1.276)	0.96
Substance use disorder (12 month)	0.892 (0.672–1.196)	0.43	0.853 (0.682–1.073)	0.17
Social media users	1.688 (1.344–2.116)	<0.0001	1.149 (0.943–1.395)	0.17

The completion rate of the daily EMA was higher in patients with major depressive disorder and bipolar disorder (mainly type 2: 0.84 ± 0.08 and 0.89 ± 0.06 vs. in patients without), but not with a current major depressive episode. Results were heterogeneous for patients with anxiety disorders: completion rate for the daily EMAs tended to be higher in patients with panic disorder (0.88 ± 0.06 vs. 0.80 ± 0.09), and lower in patients with current general anxiety disorder. No associations were observed between actual PTSD and alcohol or substance use disorders (Table 2).

Finally, the completion rate of the daily EMA (0.85 ± 0.08) was higher in social media users (Facebook, Twitter, and Instagram).

Multivariate analysis of the influence of the most significant clinical characteristics on the daily completion rates (Table 3) showed effects for all variables, but not for sex and suicidal ideation (CSSRS score at inclusion). The absence of effect could be explained by the fact that sex and suicidal ideation were strongly correlated with the depression level at inclusion. The other clinical characteristics had a specific, independent effect on the daily completion rates.

**TABLE 3:** Multivariate analysis for the daily completion rates.

Clinical characteristic	Odd ratio and 95% CI	*Z*-value	*P*-value
Women	0.95 (0.63–1.42)	−0.261	0.79
Depression (QIDS score at inclusion)	1.05 (1.02–1.08)	3.462	0.0005
Suicidal ideation (CSSRS score at inclusion)	0.76 (0.48–1.18)	−1.203	0.23
Type 2 bipolar disorder	2.17 (1.28–3.84)	2.775	0.006
Current general anxiety disorder	0.66 (0.52–0.85)	−3.286	0.001
Lifetime panic disorder	1.45 (1.04–2.05)	2.159	0.03
Social media usage	1.94 (1.45–2.58)	4.469	<0.0001

Concerning EMI utilization in the first month, the call module was used by eight patients to reach the SAMU (mean number of times ± SD and min–max, 1.5 ± 1.07, 1–4), by 12 patients to call a relative (2.25 ± 1.29, 1–5), and by 12 patients to call the emergency department (1.75 ± 0.97, 1–3). The “Breathing Space” module was used by eight patients (1.5 ± 0.71, 1–2).

### Satisfaction

Forty-five patients filled in the first monthly questionnaire with heterogenous completion rates, depending on the question. Satisfaction with *emma* was evaluated based on the response (on a Likert scale from 0 to 10, 0 being the worst score and 10 the best) to one of the most answered questions (*n* = 24/45) in the monthly EMA: “Would you recommend *emma* to friends with the same problems as you?” and was (mean and standard deviation) 6.92 ± 2.78.

Then, the global satisfaction during the last visit at M6 (*n* = 46) was evaluated with the MARS adapted to *emma*. The mean score and standard deviation (ranging from 0 to 5, 0 being the worst and 5 the best) were calculated for specific MARS sections and questions that were considered to be the most relevant for the study objective (Table 4).

**TABLE 4:** Satisfaction scores from the MARS scale.

Section A on Engagement (fun, interesting, customizable, interactive, well targeted to audience)	3.18 ± 0.88
Section B on Functionality (application functioning, easy to use, navigation, flow logic, and gestural design)	4.08 ± 0.84
Section C on Aesthetics (graphic design, overall visual appeal, color scheme, consistent style)	3.99 ± 0.73
Section D on Information (quality of information)	3.85 ± 0.85
Section E on Subjective dimensions “Would you recommend this app to people who might benefit from it?” “How many would you use this app in the next 12 months if it were relevant to you?” “What is your overall star rating of the app?”	3.85 ± 1.3 2.72 ± 0.87 3.3 ± 0.87
Section F on the *emma* application “Awareness: This app is likely to increase awareness on the importance of addressing suicidal thoughts or behavior” “Help seeking: Use of this app is likely to encourage help seeking in case of need”	3.04 ± 1.26 3.48 ± 1.43

## Discussion

Our study results suggest promising *emma* acceptance and use by the targeted audience. Completion rates of the daily EMAs were significantly associated with higher depression levels, suicidal ideation, depressive disorder, and type 2 bipolar disorder, indicating that this type of digital assessment tool is relevant for the high-risk populations who need it most.

Social media users also presented very-high completion rates. Previous studies have already showed that social media are becoming a new place where patients send their first distress signals while not doing it offline (30). Therefore, future studies should not overlook the collection of this new kind of valuable data, and might also integrate passive EMAs by precisely accessing social media usage. It would also be relevant to study whether the increased completion of the questionnaires among this typology of patients is (1) specifically related to skills they may have acquired, related to the communicative characteristics of social networks (such as new possibilities to express their distress) or (2) depends more globally on their higher literacy levels.

Our results show that patients in need were satisfied with *emma* and that this application increased their awareness on the necessity to address suicidal thoughts and behaviors. Moreover, users were inclined to recommend the application to other people in the same situation.

However, the EMA completion rate was very heterogeneous. The number of daily EMAs in the first 3 days did not suit the users who switched to a less demanding format with weekly assessments. Asking the right question at the right moment is crucial for the patients’ proper follow-up. Randomized questions could have avoided monotony and repetition bias, but the clinical logic (asking for mood or psychological pain before suicidal thoughts, asking “important” questions, such as suicidal behaviors before the secondary question on emotions or relationships, etc.) required a particular order.

Our study contains several limitations, such as the non-randomized aspect of the sample, the small sample sizes for some of our primary outcomes (e.g., *n* = 24 for the overall *emma* satisfaction score at M1, *n* = 46 for overall *emma* global satisfaction at M6), and the potential fact that patients willing to complete the follow-up visits could be more likely to feel positively about the app. But our main study limitation is the dropout rate, as previously reported for the vast majority of mental health applications that fail to gain any kind of traction, compliance, and observance (31). Several studies showed that the user base’s attrition rate is almost always high (up to 50%) before or in the first month of the experiment (32, 33). This rapid decrease in the use of digital tools is so frequent, even in clinical protocols where the subjects are voluntary, that Eysenbach proposed to theorize this phenomenon as “attrition science” (34). A recent systematic and meta-analytic review by Linardon et al. also points out that smartphone interventions evaluated by randomized controlled trials (RCTs) for psychosomatic disorders are characterized by high dropout rates and low adherence (35). We can hypothesize that the use of the application drops even more dramatically, from day 1, outside of a singular clinical trial context (36). Several explanations can be put forward.

(1) We found that one of the primary issues with EMA is the burden it may place on patients. The EMA approach requires a non-negligible amount of time and dedication from each participant.
(2) Patients tend to quickly discontinue the daily questionnaires and show a real fatigability, thus explaining why completion rates were higher for the weekly than daily EMAs. Therefore, we might need to develop more flexible and tailored EMAs for suicidal patients that would adapt and maybe only address the most relevant or changing elements. To promote user adherence, different ways of supporting and stimulating the participant-application interaction should be considered, from the very beginning of their “encounter.” This can be for example personalized feedback, entertainment, or gamification (36–39).
(3) Moreover, the multiplication of reminders (*via* notifications) could be seen as invasive and bother patients, especially if they are expected to read sensitive information or complete a task at an inconvenient time. This issue might be solved by passive and continuous data collection through connected sensors, wearables, or a smartphone that patients have always with them and that perform passive EMAs in the background, leading to a more quantitative and objective data stream. At first glance, these high attrition rates could hamper the evaluation of mental health applications. However, digital tools can quickly adapt and evolve. This means that clinicians have to anticipate this phenomenon and work to find incentives to increase the patients’ engagement.
(4) Moreover, the number of dropouts might increase progressively over time because patients tend to use the application at times of crisis and then to forget it, presumably as a result of clinical improvement and resolution of the suicidal crisis. As our study was oriented toward suicide prevention, we have to consider the flow of a suicidal crisis and the different needs during this period. Most suicide re-attempts take place in the first 6 months (40–43), and the first 1–2 weeks after the attempt are the most critical period (44). Therefore, we could couple *emma* with already existing brief contact intervention protocols that combine phone calls, postcards, and emergency numbers to reduce suicide attempts (e.g., VigilanS protocol in France) (45, 46), particularly in the first month.
(5) Lastly, we would like to reflect on what the application brings to patients besides its usage. Indeed, despite a decline in use over time, satisfaction was globally high and encouraging. Many users said that they would recommend the application, and more importantly that they would seek help in case of crisis. This shows that *emma* can achieve this connectedness it strived to develop, one of the most critical protective factors in suicidal behaviors. This opens the door for a new sociotechnical system where patients, their relatives, their caregivers, and the technology can work together to help the patient. Patients might not stick with the application, uninstall it earlier than recommended, or not use it fully. However, being accompanied by a health professional during the process and learning that these kinds of resources exist, what they represent, and the assistance they can provide is an obvious benefice. Thus, beyond the objective link that suicidal individuals may have with the *emma* application, a form of satisfaction seems to emerge from their engagement with the digital tool. This relational engagement conveys strength and vitality that encourages them to modify their thoughts, their behaviors, and more deeply their being in the world. One of the participants thus stated: “*I realized what it did, what it provoked in me, that it allowed me to move forward, to understand and to change my perspective.*” We strongly believe that through the different EMIs proposed during their time with the application patients acquire tools and learn how to help themselves, and also understand when to reach for help in case of suicidal ideation. By developing reflexivity, these applications constitute an additional way for people with suicidal behaviors to become empowered and take care of their mental health. As they represent support that is always within their reach and available, they are free to come back to it whenever they need it. Consequently, its non-usage could possibly reflect a change in the patient’s mindset and needs. Precautions are however necessary while considering possible clinical applications as even the daily EMAs presented here only represent 3 days of a month thus considerably limiting the opportunity for patients to engage in EMIs. During the first month, 32 patients found themselves in such a distressing situation that they used the “Call Module.” Most of them (20) asked for professional help *via* the application (SAMU and emergency department), while 12 were able to contact relatives they had identified as resource persons. In view of the very strong personal and structural barriers to help-seeking, *emma* seems to have played a useful role in connecting people in distress with different types of help. In contrast, only eight patients used the “Breathing Space” module, as this space probably did not meet their needs at this critical time.

These applications represent an additional tool to help patients and clinicians work together in a new paradigm in which what matters most for patients (loss of meaning, social isolation, disability associated with symptoms, etc.) is also what matters most to clinicians. As such, the use of Patient Reported Outcome Measures (PROMs) seems a useful framework to mobilize (47, 48). As we strongly believe that an application should not replace any clinician, it seems essential to us to increase literacy in the general population, people with disorders, and health professionals on the interests and limitations of these tools. Further studies could thus also focus on the mindset a patient could be in when downloading a medical application, for example, assessing if downloading an application would possibly discourage them from seeking treatment directly with a clinician. Additional research is also needed to address the attrition rate, boost engagement, and determine the real patients’ needs in digital health.

## Conclusion

As suicidal and depressed patients tended to use *emma* more often, *emma* reached its goal of targeting patients at high risk of suicide with promising levels of satisfaction. These findings fit in the current perspective of more personalized medicines.

mHealth has the potential to reduce the wide service gap in the care of people with a mental health problem (9, 10, 49) and to become a great vector for therapeutic education and patient empowerment. mHealth tools are widely available, cheap, and can be used to create a clinical database that might lead to the development of more efficient smartphone applications for mental health by enhancing their content (from self-monitoring to emotional self-awareness) and forging a better therapeutic alliance (50). Despite the currently high drop-out rate, mHealth has the potential to rapidly evolve and innovate to bring incentives and achieve better acceptability and usage.

In the context of individuals with different levels of suicidality, up to and including suicidal crisis, the effectiveness of the digital tools is particularly critical. High-level clinical studies are thus required to ensure their effectiveness in the natural social setting of these individuals, and not only in the singular setting of standardized research protocols. Nevertheless, based on the results of this study, a gap seems to exist between the objective uses of the application and the necessarily subjective feelings of the participants. Quantitative studies thus seem insufficient to capture what, in each singular assembly of individual and digital tool, has the power to transform the perception that individuals have of themselves and their practices. Complementary approaches from the social sciences are thus called for.

## Data availability statement

The raw data supporting the conclusions of this article will be made available by the authors, without undue reservation.

## Ethics statement

The studies involving human participants were reviewed and approved by the Comité de protection des personnes “EST IV.” The patients/participants provided their written informed consent to participate in this study.

## Author contributions

MM and CG: conception and design of the work, digital tool design, supervision of the study follow-up, data analysis and interpretation, and drafting the article. DY: data analysis and interpretation and drafting the article. JD and JA: data analysis and interpretation and critical revision of the article. ML: critical revision of the article. GV and SB: supervision of the study follow-up and critical revision of the article. PC: conception and design of the work, supervision of the study follow-up, and critical revision of the article. All authors contributed to the article and approved the submitted version.

## Footnotes

1
https://clinicaltrials.gov/
